# Evaluating effectiveness of cadaveric arthroscopic training for orthopaedic residents: A comparison of joints and training levels

**DOI:** 10.1002/jeo2.12030

**Published:** 2024-05-20

**Authors:** Hao‐Chun Chuang, Fa‐Chuan Kuan, Wei‐Ren Su, Chien‐An Shih, Chen‐Hao Chiang, Po‐Yen Ko, Chih‐Kai Hong, Yueh Chen, Kai‐Lan Hsu

**Affiliations:** ^1^ Department of Orthopaedic Surgery, National Cheng Kung University Hospital, College of Medicine National Cheng Kung University Tainan Taiwan; ^2^ Department of Biomedical Engineering National Cheng Kung University Tainan Taiwan; ^3^ Skeleton Materials and Bio‐compatibility Core Lab, Research Center of Clinical Medicine, National Cheng Kung University Hospital, College of Medicine National Cheng Kung University Tainan Taiwan; ^4^ Musculoskeletal Research Center, Innovation Headquarters National Cheng Kung University Tainan Taiwan; ^5^ Department of Orthopaedics Ditmanson Medical Foundation Chia‐Yi Christian Hospital Chiayi Taiwan; ^6^ Department of Orthopedics Kaohsiung Veterans General Hospital Tainan Branch Tainan Taiwan

**Keywords:** arthroscopy, education, resident training

## Abstract

**Purpose:**

This study aimed to evaluate the effects and interactions of training level and different joints on the outcomes of cadaveric arthroscopic training courses for orthopaedic residents.

**Methods:**

This prospective study enrolled 16 orthopaedic residents who voluntarily participated in a cadaveric training programme involving the shoulder, elbow, wrist, knee and ankle joints. Outcomes were quantitatively assessed using task‐specific checklists and the Arthroscopic Surgery Skill Evaluation Tool. Two‐way analysis of variance (ANOVA) was conducted to determine the significance of the interactions between joint and years of training.

**Results:**

Resident scores significantly increased after the dedicated lectures in all five joints (*p* = 0.003 for the shoulder module, *p* < 0.001 for the other joints). Two‐way ANOVA revealed that the progress made after the dedicated lectures was significantly impacted by the joint (*p* = 0.006) and training level × joint interaction (*p* = 0.005) but not by the training level (*p* = 0.47). The simple effect of the joint was examined using Sidak's multiple comparison test. Among junior residents, the dedicated lectures resulted in more substantial progress in elbow and wrist arthroscopy when compared to shoulder arthroscopy (*p* = 0.020 and *p* = 0.043, respectively).

**Conclusions:**

The results suggest that, in cadaveric arthroscopic training courses for orthopaedic residents, training outcomes are primarily impacted by the specific joint being trained rather than the training level. Specifically, junior residents demonstrated greater improvement with training in procedures that are less commonly encountered during on‐the‐job training, such as elbow and wrist arthroscopy.

**Clinical Relevance:**

These findings suggest the need to prioritise wrist and elbow arthroscopic training for junior residents to optimise educational outcomes.

**Level of Evidence:**

Level III.

**Abbreviation**
PGY: postgraduate year

## INTRODUCTION

Arthroscopy has a steep learning curve and requires the development of sophisticated psychomotor skills; thus, participation in arthroscopic surgeries has become an integral part of orthopaedic residency training [25]. Conventionally, arthroscopic techniques were learned during on‐the‐job training. Despite increases in the overall arthroscopic caseload among orthopaedic residents, their experience performing arthroscopic procedures varies substantially [12, 14]. For instance, some studies showed that residents' exposure to ankle arthroscopy remained low in recent years, indicating that reliance solely on on‐the‐job training might leave a gap in resident training [32]. Some resident training programmes introduced surgical skill laboratories and simulation technology to help achieve technical competence [21, 22]. However, the simulators are costly, limited joints can be simulated and their transferability to clinically applicable skills remains unsubstantiated [10, 16, 25, 27]. The most realistic training programmes still employ cadaveric models for joint‐specific arthroscopy simulations [1, 13]. The addition of cadaver laboratory training in knee arthroscopy reportedly improved residents' surgical techniques and benefited their overall education [9, 19]. However, cadaveric simulation training is not necessarily more accessible than simulator training. This is because most cadaveric courses are not freely available, and each specimen can only be used once. Additionally, ethical and safety concerns often accompany cadaveric training [6]. Proper allocation of cadaveric educational resources is, therefore, a problem worth considering.

Aside from the educational benefits of cadaveric or simulated arthroscopy, a growing number of studies have analysed factors affecting the efficiency of arthroscopic training courses [13, 15, 22]. In shoulder, knee and ankle arthroscopy, a positive correlation was found between training level (postgraduate year [PGY]) and performance [15, 22]. Sleep deprivation, in contrast, negatively affects the outcome of simulator training for residents [3]. Interestingly, Henn et al. found that simulation training in knee arthroscopy did not improve proficiency in wrist arthroscopy among residents, indicating that competence in arthroscopy was not transferrable between joints, and a joint‐specific arthroscopy training programme is needed [13]. Currently, there is a lack of studies comparing the effects of cadaveric arthroscopic training courses across various joints.

To bridge the gap between past studies, the current study was designed to simultaneously explore the effect of training level and different joints on the learning outcomes. Junior and senior orthopaedic residents were recruited to a multijoint cadaveric training workshop. It was hypothesised that both training level and different joints were associated with learning outcome. Additionally, an interaction between the training level and joint was also hypothesised. The results will help resident course directors tailor curricula to meet residents' needs and thereby maximise the efficacy of educational resources.

## METHODS

### Population

The study included a prospective single institutional cohort of 16 PGY 2–6 orthopaedic residents who voluntarily participated in the cadaveric training programme. The mean age of the participants was 28 years (range, 25–31 years), with 14 male and two female participants. Three participants had previously received cadaveric training, while the rest were cadaveric training naïve. This research was conducted at a tertiary care centre in southern Taiwan. The hospital had a surgical training suite that regularly offered cadaveric training courses for different levels. In the residency programme, junior residents (PGY 2–3) have 8–12 weeks and senior residents have 4–8 weeks of sports medicine rotations per year. Each rotation consisted of weekly clinical case conferences and didactic lectures. The surgical cases included both arthroscopic and open procedures. In addition to standard on‐the‐job training, resident trainees could also practice arthroscopic skills using a shoulder simulator model in the education laboratory.

### Workshop scheduling

The participants in this study completed an intensive 1‐day cadaveric training course involving the use of fresh‐frozen cadaveric joints from the shoulder, elbow, wrist, knee and ankle, which were set up on operating tables at five stations. Commercially procured fresh‐frozen human cadaveric material was used (Science Care Ltd.). The participants were divided into groups of three and rotated through the stations. At each station, all participants performed a diagnostic arthroscopy test, rotating roles as the first surgeon, assistant and scrub nurse. The procedures were supervised and timed by the attending faculty.

During the initial rotation through the stations, the participants performed diagnostic arthroscopy based on their previous on‐the‐job training without a didactic lecture. Following this, the participants completed a competency‐based training, which included 3 h of didactic lectures on arthroscopic techniques and surgical approaches. After the lectures, the participants immediately retook the previously completed test using a new set of cadaveric joints.

### Evaluations

Resident evaluations were conducted using standardised cadaveric testing systems based on task‐specific checklists (Supporting Information) that were developed from previously validated checklists [4, 8, 13, 15, 18, 24, 26, 27], and integrated aspects of the Arthroscopic Surgery Skill Evaluation Tool [28]. The checklists included items on portal placement, diagnostic arthroscopy and arthroscopic anatomy. In developing the checklists, sports medicine specialists consulted established ones and identified six clinically relevant anatomical landmarks. To simulate real operating theatre conditions and streamline workshop proceedings, the number of tasks was limited to ensure completion within 3 min per operator. Within each joint, six tasks were assigned, with an anticipated completion time of 30 s per task. Senior orthopaedic surgeons from different subspecialties, excluding sports medicine, reviewed the checklists to assess feasibility. Benchmark times were established for each subcategory. The procedures were supervised by attending surgeons in orthopaedic sports medicine.

Before the initial rotation, the participating residents were provided with an outline of the tasks to be completed and the anatomical structures to be identified during the procedure, with the expectation that they would follow it without requiring consent or interference from the attending surgeon. At the start of the assessment, the residents were reminded to follow the steps listed on the given checklist. The supervising attending surgeon used a digital timer to measure the duration of each procedure and assessed the procedure using a standardised checklist on a scale from 0 to 12. The residents had to complete the tasks sequentially and could not proceed to the next checklist item unless the preceding items had been checked. The attending surgeons refrained from intervening during the procedure.

This process was repeated after the dedicated lecture. The primary intervention in this study was the engagement in the dedicated lecture and assistance during the procedure. The primary outcome measure was the number of items checked on the checklist. Pre‐ and posttraining arthroscopies were evaluated by the same attending faculty who also delivered didactic lectures on the relevant anatomical sections. Pre‐ and postlecture checklist scores were recorded by staff in an Excel spreadsheet for storage and grading purposes.

### Statistics

To determine whether the lecture had a positive impact on progress, pre‐ and postlecture scores were compared using a paired Student's *t*‐test. To evaluate the factors influencing progress, a two‐way analysis of variance (ANOVA) analysed the impact of anatomical section (knee, shoulder, elbow, wrist and ankle joints), training experience (senior versus junior) and their interaction. An interaction would manifest if the effect of training level on progress differs across anatomical sections. Put differently, the correlation between anatomical section and progress varies based on whether the resident is senior or junior. If the interaction term was statistically significant, its simple effect was tested using Sidak's multiple comparison test. Microsoft Excel version 15.0 (2013) and SPSS (version 17) (IBM) were used for the statistical analysis.

## RESULTS

### Effect of workshop on participants

All 16 residents attended the dedicated lectures and completed pre‐ and postlecture simulations. In all five joints, residents' scores significantly increased after the dedicated lectures (*p* = 0.003 for the shoulder module, *p* < 0.001 for the other joints). The mean prelecture scores for the knee, shoulder, elbow, wrist and ankle were 8.5, 9.3, 3.5, 6.5 and 2.8, respectively. The mean posttest scores for the knee, shoulder, elbow, wrist and ankle were 11.2, 11.4, 8.0, 9.9 and 6.5, respectively (Table 1A). When the attendees were stratified by seniority, the progress made by junior residents was statistically significant in all joints except the shoulder joint (Table 1B). Senior residents, in contrast, made significant progress in all joints (Table 1C).

**Table 1:** Summary of mean pre‐ and postlecture scores for each joint.

(A) Pre‐ and postlecture scores of all residents					
Total (*N* = 16)	Mean prelecture score	SD	Mean postlecture score	SD	*p* Value^a^
Knee	8.5	1.3	11.2	1.8	<0.001
Shoulder	9.3	2.6	11.4	0.8	0.003
Elbow	3.5	1.0	8	1.9	<0.001
Wrist	6.5	2.9	9.9	0.3	<0.001
Ankle	2.8	1.2	6.5	1.9	<0.001

(B) Pre‐ and postlecture scores of junior residents					
Junior (*n* = 7)	Mean prelecture score	SD	Mean postlecture score	SD	*p* Value
Knee	8.0	1.2	10.9	2.3	0.001
Shoulder	10.0	1.7	11.0	0.8	0.111
Elbow	3.1	1.2	7.4	2.7	0.006
Wrist	4.9	2.5	9.7	0.5	0.002
Ankle	3.0	1.3	5.4	1.7	0.004

(C) Pre‐ and postlecture scores of senior residents					
Senior (*n* = 9)	Mean prelecture score	SD	Mean postlecture score	SD	*p* Value
Knee	8.9	1.3	11.4	1.3	0.001
Shoulder	8.8	3.1	11.7	0.7	0.011
Elbow	3.8	0.8	8.4	1.0	<0.001
Wrist	7.8	2.5	10.0	0.0	0.030
Ankle	2.6	1.2	7.3	1.7	<0.001

Abbreviation: SD, standard deviation.

^a^ The pre‐ and postlecture scores were compared using the paired Student's *t*‐test.

### Analysis of factors influencing progress made by the residents

**Figure 1:**
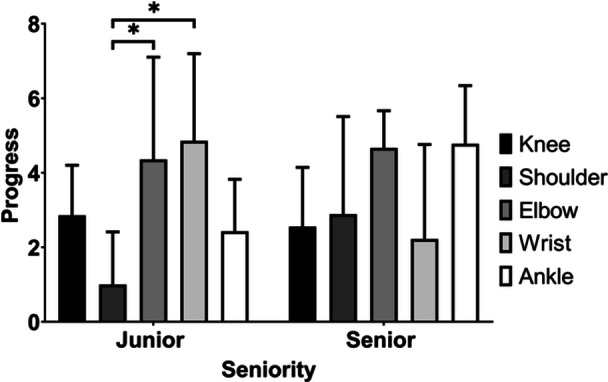
Simple effect of joint on cadaveric arthroscopic training outcomes. Among junior residents, the dedicated lectures resulted in more substantial progress in elbow and wrist arthroscopy when compared to shoulder arthroscopy (*p* = 0.020 and *p* = 0.043, respectively). Among senior residents, the improvements brought about by the dedicated lectures were not significantly different. The effect was examined by two‐way analysis of variance and post hoc Sidak's multiple comparison test.

Factors affecting the progress made were analysed using a two‐way ANOVA, which revealed a statistically significant training level × joint interaction (*p* = 0.005). The joint also had a significant main effect on progress (*p* = 0.006), while the training level did not (*p* = 0.47) (Table 2). The interaction indicated that different joints affected the learning outcomes of junior and senior residents differently. Therefore, the simple effect of the joint was examined using Sidak's multiple comparison test. Among junior residents, the dedicated lectures resulted in more substantial progress in elbow and wrist arthroscopy when compared to shoulder arthroscopy (*p* = 0.020 and *p* = 0.043, respectively). Among senior residents, the improvements provided by the dedicated lectures were not significantly different (Figure 1).

**Table 2:** Progress made by junior versus senior residents before and after dedicated lectures.^a^

	Junior	Senior	*p* Value^b^
Knee	2.9 ± 1.3	2.5 ± 1.6	Anatomical region: 0.006
Shoulder	1.0 ± 1.4	2.9 ± 2.6	Seniority: 0.467
Elbow	4.4 ± 2.7	4.7 ± 1.0	Anatomical region × seniority: 0.005
Wrist	4.9 ± 2.3	2.2 ± 2.5	
Ankle	2.4 ± 1.4	4.8 ± 1.6	

^a^ Progress was defined as the difference between pre‐ and postlecture scores and is shown as mean ± standard deviation.

^b^ Interaction and main effects were examined using two‐way analysis of variance.

## DISCUSSION

Orthopaedic residents' arthroscopic training is integral to their education, and a vast literature exists on the various modalities used for this purpose. The present study focused on arthroscopic training in cadaveric joints and is one of the few to date to compare different joints directly. In contrast to previous research [26, 28], our findings showed that training outcomes were not primarily determined by the level of training, but rather by the specific joint being trained. Specifically, junior residents demonstrated significant improvements as a result of cadaveric training in elbow and wrist arthroscopy, which are relatively less commonly encountered during on‐the‐job training. These results could aid in the organisation of training courses for orthopaedic residents and enhance the efficiency of educational resources.

Previous research compared the outcomes of arthroscopic training in different joints [26, 28]. One study found that junior‐level residents showed greater improvements in the knee simulator, while senior‐level residents showed greater improvements in the shoulder simulator [28]. Another study compared the time it took to complete arthroscopic knee and shoulder checklist items and found that the mean improvement in time was greater in shoulder simulations than in knee simulations (822.6 versus 303 s) [26]. However, these studies did not make comparisons between all commonly encountered joints, and most tested the utility of simulators rather than cadaveric arthroscopic training. The validity of arthroscopic simulators as a training tool requires further investigation before being integrated into resident training [2, 35]. To address these gaps, the current study simultaneously compared the educational outcomes of cadaveric arthroscopic training in the five most common joints. Results of the current study indicated that junior residents benefitted the most from courses in elbow and wrist arthroscopy, while the progress made by senior residents was not significantly different by joint. This difference may be due to the relatively limited exposure to elbow and wrist arthroscopic surgeries at our institution. Other institutions have also reported difficulty in accessing wrist and elbow arthroscopies for orthopaedic residents [7, 17, 33]. The limited on‐the‐job access to upper limb arthroscopies for residents highlights the importance of proper arrangement of resident training courses. Consequently, we recommend prioritising wrist and elbow arthroscopic training for junior residents to optimise educational outcomes.

While many studies have found positive correlations between seniority and surgical outcomes [30], only a few evaluated the impact of seniority on learning outcomes [3, 5, 22]. For example, Baumann et al. found that the level of seniority was strongly correlated with performance on diagnostic ankle arthroscopy in a cadaveric model and observed year‐to‐year increases in global operative skills rating scales, task‐specific checklists, and oral questionnaires [3]. Martin et al. also reported similar findings in shoulder arthroscopic training, noting a 16‐s improvement in the time required to complete a standardised object localisation task for every additional year of seniority [22]. Cannon et al. found that senior residents required significantly shorter times to complete tasks and achieved a higher degree of completeness in arthroscopic knee simulators [5]. These previous studies have largely focused on training in a single joint and have reported improvements in composite scores or time. In contrast, the current study analysed the progress made by residents in multiple joints and compared the effect of seniority on learning outcomes. By analysing progress and controlling for baseline pretest scores obtained from on‐the‐job training, the current results suggest that learning outcomes were not significantly compromised by the lack of on‐the‐job exposure. These findings suggest that incorporating cadaveric workshops into resident training may be beneficial for both junior and senior residents.

Organising and structuring the itinerary for a cadaveric training workshop requires considerable effort and attention to detail. Distributing learning sessions over time has been demonstrated to enhance long‐term retention of knowledge [31, 36]. Similarly, the spacing of pre‐ and posttraining tests has shown effectiveness for surgical technology students [11]. Mackenzie et al. even spaced the posttraining test by intervals of 1 month or 3–6 months apart [20]. Conversely, arranging pre‐ and posttraining testing on the same day offers logistical advantages and closely mirrors the hands‐on learning pattern in a real operating theatre. In a simulation course for vascular surgical skills, intensive pre‐ and posttraining tests still improved procedural knowledge and self‐rated procedural competence of participants [29]. For the sake of cadaveric specimen preservation and logistical simplicity in arranging the workshop, residents in the current study underwent pre‐ and posttraining testing on the same day. The results of the study supported the effectiveness of same‐day education and testing for the acquisition of arthroscopic techniques.

There are several limitations to this study. First, the study design is cross‐sectional, and the sample size is relatively small. To address this, we plan to conduct a longitudinal study to include more participants and to assess changes in scores of the current cohort after adequate follow‐up. Second, hip arthroscopy was not included in the study, as it is a relatively low‐volume procedure during orthopaedic residency [34]. Third, the study did not include psychomotor parameters such as total probe distance and arthroscopic tip distance. The use of motion‐tracking handles could provide an additional objective method for measuring improvement and take into consideration the level of dexterity shown by the resident or the time spent on each of the steps [23]. Hence, the current study relied on semiobjective checklist‐based scoring. Future training workshops aim to address these limitations by incorporating video records and motion‐tracking handles.

## CONCLUSION

In this study of cadaveric arthroscopic training courses for orthopaedic residents, training outcomes were primarily impacted by the specific joint being trained rather than the training level. Specifically, junior residents demonstrated greater improvement with training in procedures that are less commonly encountered during on‐the‐job training, such as elbow and wrist arthroscopy. These findings suggest the need to prioritise wrist and elbow arthroscopic training for junior residents to optimise educational outcomes.

## AUTHOR CONTRIBUTIONS

Hao‐Chun Chuang and Kai‐Lan Hsu made substantial contributions to the conception and design of the current study. Hao‐Chun Chuang, Fa‐Chuan Kuan, Wei‐Ren Su and Chih‐Kai Hong contributed to the analysis and interpretation of data for the work. Fa‐Chuan Kuan, Wei‐Ren Su, Chien‐An Shih, Chen‐Hao Chiang, Po‐Yen Ko, Chih‐Kai Hong and Yueh Chen contributed to the education, skill evaluation and data acquisition. Hao‐Chun Chuang, Fa‐Chuan Kuan, Chih‐Kai Hong and Kai‐Lan Hsu drafted the work and revised it critically for important intellectual content. Wei‐Ren Su, Chih‐Kai Hong and Kai‐Lan Hsu contributed to the final approval of the version to be published. All authors made an agreement to be accountable for all aspects of the work in ensuring that questions related to the accuracy or integrity of any part of the work are appropriately investigated and resolved.

## CONFLICT OF INTEREST STATEMENT

The authors declare no conflict of interest.

## ETHICS STATEMENT

The institutional review board at the National Cheng Kung University Hospital approved this cadaver use (B‐ER‐108‐170).

## Supporting information

Supporting Information.

## Data Availability

The data that support the findings of this study are openly available.
