# Supplementary material for: Evaluating effectiveness of cadaveric arthroscopic training for orthopaedic residents: A comparison of joints and training levels
Source: J Exp Orthop. 2024 May 20;11(3):e12030. doi: 10.1002/jeo2.12030 (PMC11106551; doi:10.1002/jeo2.12030)
Supplement: Supplementary file 1 — Supporting Information. [file JEO2-11-e12030-s001.docx]

**Supplementary files: Task-specific checklists used in the current study**

Task-specific checklist: diagnostic arthroscopy of the knee

| **Task** | **Complete (points)** |
| --- | --- |
| Inspect suprapatellar pouch and patellofemoral joint | 🞎Yes (2) 🞎Partially (1) 🞎No (0) |
| Inspect the lateral and medial gutter and probe femoral condyles and tibial plateau | 🞎Yes (2) 🞎Partially (1) 🞎No (0) |
| Inspect and probe the medial and lateral menisci | 🞎Yes (2) 🞎Partially (1) 🞎No (0) |
| Inspect and probe ACL and PCL via anterior portals | 🞎Yes (2) 🞎Partially (1) 🞎No (0) |
| Inspect the popliteus tendon and recess | 🞎Yes (2) 🞎Partially (1) 🞎No (0) |
| Inspect and probe the PCL insertion via posterior portals | 🞎Yes (2) 🞎Partially (1) 🞎No (0) |

ACL, anterior cruciate ligament; PCL, posterior cruciate ligament

Task-specific checklist: Diagnostic arthroscopy of the shoulder

| **Task** | **Complete (points)** |
| --- | --- |
| Inspect and probe the bicipital long head tendon | 🞎Yes (2) 🞎Partially (1) 🞎No (0) |
| Inspect and probe the subscapularis tendon | 🞎Yes (2) 🞎Partially (1) 🞎No (0) |
| Inspect and probe the superior and anterior labrum | 🞎Yes (2) 🞎Partially (1) 🞎No (0) |
| Inspect and probe the anterior band of the inferior glenohumeral ligament | 🞎Yes (2) 🞎Partially (1) 🞎No (0) |
| Switch the viewing and working portals to probe the posterior labrum | 🞎Yes (2) 🞎Partially (1) 🞎No (0) |
| Inspect and probe the acromial undersurface across the subacromial space | 🞎Yes (2) 🞎Partially (1) 🞎No (0) |

Task-specific checklist: Diagnostic arthroscopy of the elbow

| **Task** | **Complete (points)** |
| --- | --- |
| Establish the anteromedial and anterolateral portals | 🞎Yes (2) 🞎Partially (1) 🞎No (0) |
| Inspect and probe the trochlea and capitellum | 🞎Yes (2) 🞎Partially (1) 🞎No (0) |
| Inspect and probe the radial head and annular ligament | 🞎Yes (2) 🞎Partially (1) 🞎No (0) |
| Inspect and probe the coronoid process | 🞎Yes (2) 🞎Partially (1) 🞎No (0) |
| Inspect and probe the junction of the anterior joint capsule and distal humerus | 🞎Yes (2) 🞎Partially (1) 🞎No (0) |
| Inspect and probe the olecranon and olecranon fossa | 🞎Yes (2) 🞎Partially (1) 🞎No (0) |

Task-specific checklist: Diagnostic arthroscopy of the ankle

| **Task** | **Complete (points)** |
| --- | --- |
| Evaluate anteromedial gutter | 🞎Yes (2) 🞎Partially (1) 🞎No (0) |
| Inspect and probe the AITFL | 🞎Yes (2) 🞎Partially (1) 🞎No (0) |
| Inspect and probe the syndesmosis | 🞎Yes (2) 🞎Partially (1) 🞎No (0) |
| Inspect and probe the PITFL | 🞎Yes (2) 🞎Partially (1) 🞎No (0) |
| Inspect and probe the ATFL | 🞎Yes (2) 🞎Partially (1) 🞎No (0) |
| Switch to the posterior portals to probe the posterior talar process and FHL | 🞎Yes (2) 🞎Partially (1) 🞎No (0) |

AITFL, anterior inferior tibiofibular ligament; ATFL, anterior talo-fibular ligament; FHL, flexor hallucis longus; PITFL, posterior inferior tibiofibular ligament

Task-specific checklist: Diagnostic arthroscopy of the wrist

| **Task** | **Complete (points)** |
| --- | --- |
| Establish the 3,4 viewing portal and 6R portal | 🞎Yes (2) 🞎Partially (1) 🞎No (0) |
| Inspect and probe the radio-lunate fossa and radio-scaphoid fossa | 🞎Yes (2) 🞎Partially (1) 🞎No (0) |
| Inspect and probe the radio-lunate ligaments and radioscaphocapitate ligament | 🞎Yes (2) 🞎Partially (1) 🞎No (0) |
| Inspect and probe the scapho-lunate and luno-triquetral ligaments | 🞎Yes (2) 🞎Partially (1) 🞎No (0) |
| Inspect and probe the ulnar pre-styloid recess | 🞎Yes (2) 🞎Partially (1) 🞎No (0) |
| Inspect and probe the TFCC and perform the trampoline test | 🞎Yes (2) 🞎Partially (1) 🞎No (0) |

TFCC, triangular fibrocartilage complex
